# Heat of Combustion of Borazine, B_3_N_3_H_6_

**DOI:** 10.6028/jres.065A.012

**Published:** 1961-04-01

**Authors:** Marthada V. Kilday, Walter H. Johnson, Edward J. Prosen

## Abstract

The heat of combustion of liquid borazine has been determined according to the following equation:

$$\begin{array}{l} B_{3}N_{3}H_{6}\left(\mathrm{liq}\right)+15/4\;O_{2}\left(g\right)+3/2\;H_{2}O\;\left(\mathrm{liq}\right)=3H_{3}{\mathrm{BO}}_{3}\left(c\right)+3/2N_{2}\left(g\right),\\ \Delta H{}^{\circ}\left(25{}^{\circ}C\right)=-2313.3\pm 12.6\;\mathrm{kj}/\mathrm{mole}\;\left(-552.90\pm 3.0\;\mathrm{kcal}/\mathrm{mole}\right). \end{array}$$

From this value the heat of formation of liquid borazine may be calculated as Δ*Hf°*(25 °C) = −548.5 ± 13.4 kj/mole (−131.1 ± 3.2 kcal/mole), and for the gas, Δ*Hf°*(25 °C) = −519.2 ± 13.4 kj/mole (−124.1 ± 3.2 kcal/mole).

## 1. Introduction

The determination of the heat of combustion of borazine, or borazole, was undertaken as part of the Bureau’s program for determining thermochemical properties of boron compounds. Several compounds containing the borazine ring have been prepared [1, 2, 3, 4, 5],^1^ but few thermochemical data have been reported for them [6, 7, 8, 9]; no data on the heat of formation of borazine itself have been reported.

Liquid borazine of high purity was burned in an oxygen atmosphere in a bomb, forming boric acid, boron nitride, and gaseous nitrogen as products. From the results of these combustion experiments a value for the heat of formation of borazine was calculated.

## 2. Materials and Apparatus

The borazine sample, supplied by the Naval Research Laboratory, was prepared by reducing trichloroborazole with lithium borohydride in ether solution. Details of the method are described by Shaeffer and coworkers [10]. The purity^2^ of the sample was 99.5 mole percent. No attempt was made to correct the calorimetric results for the 0.5 percent impurity in the sample; the impurity was not identified, but was believed to be a derivative of ether with a boiling point near that of borazine.

The calorimeter was the isothermal-jacket type generally used for bomb calorimetry; the temperature of the jacket was maintained within ± 0.001 °C at approximately 28 °C. A general description of the jacket, thermometric system, and bomb is given in [11]. Temperature measurements were made with a platinum resistance thermometer in conjunction with a Mueller G–2 bridge. The modified Parr bomb (vol. 376 ml) was sealed with a gold gasket and equipped with twin valves for convenience in flushing before filling with oxygen and in removing the gaseous products of combustion. Commercial oxygen used in filling the bomb was purified by passing through a furnace containing copper oxide at 600 °C to remove combustible materials, and then through an absorber containing Ascarite to remove carbon dioxide.

## 3. Experimental Procedure

The borazine samples, 0.5 to 1.0 g, were transferred in the vapor phase at room temperature into spherical soft-glass bulbs cooled to about −5 °C with an ice-salt mixture. The filled bulbs were sealed under vacuum, weighed carefully, and stored at −15 °C. In the first experiment, the sample was enclosed in a Pyrex-glass bulb and ignited by means of a platinum fuse wire instead of the usual iron wire, to eliminate possible complications from the iron oxides during analysis of the products; thereafter, iron fuse wire was used. Pyrex-glass bulbs were unsatisfactory for this work because only a small area of the glass was softened upon ignition, and incomplete combustion resulted. Soft-glass bulbs usually shattered upon ignition and the sample burned with free access to oxygen.

In assembling the bomb, the sample bulb was placed in a quartz crucible, 3 cm in diameter and 1.5 cm deep, which was supported a little below the center of the bomb. The carefully weighed fuse-wire of pure iron, 5 cm long and 5 mils in diameter, used for igniting the sample was placed in direct contact with the sample bulb. After 2 ml of distilled water had been added to the bomb, it was closed and flushed to displace air before filling with oxygen to a pressure of 30 atm. The bomb was placed in a calorimeter can containing 2968 g of water, the standard amount for the system.

The energy equivalent of the calorimetric system was determined in calibration experiments in which benzoic acid, NBS Standard Sample 39g, was burned. The heat evolved in the combustion of this sample is certified to be 26.4338 ± 0.0026 kj/g at the standard bomb conditions.

Temperature observations during the experiments were divided into four groups, an initial rating period (20 min), a combustion period (5 min), an equilibration period (11 min), and a final rating period (20 min). The rapid temperature change during the combustion period was recorded by means of a chronograph (Gaertner Scientific Co.) having a synchronous motor and two pens marking a waxed tape which advanced uniformly at 10 mm per sec. One pen recorded time in seconds as obtained from standard signals transmitted by the National Bureau of Standards, the other recorded the times when a telegraph key was tapped indicating when the resistance of the thermometer in the calorimeter passed certain previously selected values. Temperatures were recorded at 1-min intervals during the equilibration period, and at 2-min intervals during the rating periods.

The gaseous products of the experiments reported here were not analysed since nitrogen oxides were not detected in any of the preliminary experiments in which the product gases were bubbled through distilled water and the solution titrated with 0.1-*N* sodium hydroxide.

The solid products remaining in the bomb were washed with hot distilled water; the washings were filtered to remove fragments of the shattered crucible, glass bulb, and undissolved products of combustion. The filtrate, containing nitric and boric acids, was titrated with 0.1-*N* sodium hydroxide, using a Beckmann *p*H meter to determine the end points. The amount of nitric acid present was determined from the first titration curve; d-mannitol was then added to the solution to form a complex with the boric acid and a decrease in the *p*H resulted; the amount of boric acid was determined from the second titration curve. No method was found for making a quantitative separation of the insoluble products and glass fragments.

## 4. Data and Discussion

The atomic weights used are given in the International Table of Atomic Weights [12]. All heat capacity data were taken from [13] except that of borazine. No values for the density and heat capacity of liquid borazine were available, so the corresponding values for liquid benzene were used, i.e., *d*_28_ = 0.871 g/cm^3^ [14] and *Cp*(28 °C) = 32.5 cal/mole °C or 16.7 j/g-ohm [15]. The unit of energy was the joule, and for conversion to the thermochemical calorie the following relation was used: 1 thermochemical calorie = 4.1840 joules.

The products of combustion of borazine contained grey-white material which was insoluble in water and which was not attacked during digestion in aqua regia; the material was assumed to be boron nitride. This assumption was supported by an X-ray diffraction analysis^3^ although positive identification was not possible because the X-ray powder pattern was not clearly defined.

Elemental boron was not present in the products of combustion in detectable quantity. The insoluble residue of combustion was digested in aqua regia for several hours to oxidize the boron to boric acid. The solution was then filtered, and the filtrate was neutralized with sodium hydroxide, made slightly acid, and titrated to the first end point. When d-mannitol was added, no significant decrease in *p*H occurred indicating that boric acid (or boron) was not present. Since elemental boron was not found, it was assumed that any boron in the sample which was not titrated as boric acid in the water soluble products remained in the insoluble residue as boron nitride; furthermore, it was assumed that any nitrogen in the borazine sample which was not accounted for as nitric acid or boron nitride, was present in the products as gaseous nitrogen. Nitrogen oxides were not found in the gaseous products of preliminary experiments.

Data for the borazine combustion experiments are given in table 1. The amounts of nitric acid and of boric acid found in titrations are given in the second and third columns. The ratio of boric acid titrated to the boric acid calculated from the weight of sample may be used as an indication of completeness of combustion. BN is the amount of boron nitride in the products (by assumption) ; it is the difference between the number of moles of boron in the sample and the number of moles of boron titrated as boric acid.

The energy equivalent for the calorimetric system was 141,715.1 ± 9.2 j/ohm for a temperature rise of 1 °C. Heat-capacity corrections for deviations from the calibrated system are included in Δ*e* (table 1); *E_a_* is the energy equivalent of the actual system used; and Δ*Rc* is the temperature rise corrected for cooling and stirring energies as described by Prosen [16]. The ignition energy, *q_i_*, is equal to 7.50 kj/g of iron fuse-wire. (The ignition energy for the platinum fuse-wire used in expt. 1 was too small to measure.) The correction for the formation of nitric acid, *q_n_*, is 57.8 kj/mole; W.C., the Washburn correction, was calculated as described in [16, 17]. W.C. for experiments 1 and 3 also includes corrections of −0.7 *j* and −0.1 *j*, respectively, for deviations from 28 °C, the temperature of the isothermal reaction. In table 1, *S* is the weight in vacuo of the sample.

The energy for converting the boron nitride to boric acid and gaseous nitrogen, *q*_BN_, was calculated from the value for the combustion of boron nitride [18, 19], according to equation (1).

BN(c)+34O2(g)+32H2O(liq)=H3BO3(c)+12N2(g),ΔH(25°C)=−99.3kcal/mole. (1)

This is equivalent to 415 kj/mole of BN for the constant volume process at 28 °C.

The heat of combustion of borazine for the constant-volume process in which the products and reactants are in their thermodynamic standard states was calculated according to the following equation:

$$-\Delta E{}^{\circ}(28\;{}^{\circ}C)=\frac{E_{a}(\Delta Rc)-q_{i}-q_{n}+W.C.+q_{\mathrm{BN}}}{80.532\;S}.$$

The mean heat of combustion, Δ*E°*(28 °C), was −2307.91 ± 2.26 kj/mole or −551.60 ± 0.54 kcal/mole for the reaction given in equation (2).

B3N3H6(liq)+/154O2(g)+3/2H2O(liq)=3H3BO3(c)+32N2(g) (2)

The heat of combustion at constant pressure, or enthalpy change, is obtained by adding Δ*nRT*= −1.35 kcal/mole; thus, Δ*H°*(28 *°*C) = −552.95 kcal/mole. By adding a correction of 0.05 kcal/mole for the change in temperature, we obtain Δ*H°* (25 °C) = −552.90 ± 3.0 kcal/mole. The uncertainty assigned to this value is the square root of the sum of the squares of the individual uncertainties. Included in this uncertainty are twice the standard deviations of the means of the combustion experiments and of the calibration experiments, an estimated uncertainty of 0.1 percent for weights and for titrations, and 0.5 percent estimated error from sample impurity and from the uncertainty in final state of boric acid. The latter value was assigned arbitrarily because the impurity in the sample was not identified and the amount of boric oxide in the products of combustion was not determined. It is believed that the boron in the sample is burned to boric oxide which is then hydrated to boric acid. Two-thirds of this hydration probably occurs with water formed by combustion of the hydrogen in the sample. The remaining water of hydration must be supplied from the water vapor in the bomb atmosphere which is saturated at the beginning and end of the experiment; an error of as much as 7 kcal/mole would be introduced if no water reacted with the boric oxide. However, the amount of boric oxide remaining at the end of an experiment was probably very small because the solid products were quite evenly distributed over all the inner surfaces of the bomb providing maximum surface for the hydration reaction to take place. A relatively small uncertainty exists regarding the amount and concentration of boric acid in solution. This error is in the opposite direction and tends to cancel the error due to incomplete hydration of boric oxide.

The heat of formation of crystalline boric acid, −262.16 ± 0.32 kcal/mole [19], and of liquid water, −68.3174 ± 0.0100 kcal/mole [13], were used to calculate the heat of formation of liquid borazine,

$$\begin{array}{l} \Delta Hf{}^{\circ}\left(25\;{}^{\circ}C\right)=-548.5\pm 13.4\;\mathrm{kj}/\mathrm{mole}\;\text{or}\\ \;-131.1\pm 3.2\;\mathrm{kcal}/\mathrm{mole}. \end{array}$$

The heat of vaporization of borazine was obtained from the data of Stock, Wiberg, and coworkers [7, 8, 9]. A least squares fit of their data yields the following equation for the vapor pressure,

$$\log\;p_{mm}=6.39278-\frac{919.131}{\left(T-67.198\right)};$$

this may be used with the Clausius-Clapeyron equation to obtain

$$\Delta H_{v}\;(25\;{}^{\circ}C)=7.01\;\mathrm{kcal}/\mathrm{mole}.$$

Thus, the heat of formation of borazine gas is

$$\begin{array}{l} \Delta Hf{}^{\circ}\left(25\;{}^{\circ}C\right)B_{3}N_{3}H_{6}\left(g\right)=-519.2\pm 13.4\;\mathrm{kj}/\mathrm{mole}\;\text{or}\\ \;-124.1\pm 3.2\;\mathrm{kcal}/\mathrm{mole}. \end{array}$$

## 5. Summary

The heat of combustion at constant pressure was determined by burning liquid borazine in oxygen in a bomb calorimeter. For the reaction,

B3N3H6(liq)+154O2(g)+32H2O(liq)=3H3BO3(c)+32N2(g),

it was found that Δ*H°* (25 °C) = −552.90 ± 3.0 kcal/mole. From this value the heat of formation of liquid borazine was calculated to be Δ*Hf*°(25 °C) = −548.5 ± 13.4 kj/mole or −131.1 ± 3.2 kcal/mole, and that for gaseous borazine to be

$$\begin{array}{l} \Delta Hf{}^{\circ}\left(25\;{}^{\circ}C\right)=-519.2\pm 13.4\;\mathrm{kj}/\mathrm{mole}\;\text{or}\\ \;-124.1\pm 3.2\;\mathrm{kcal}/\mathrm{mole}. \end{array}$$

We wish to thank R. R. Miller of the Naval Research Laboratory for supplying the sample of borazine used in these determinations.

## Figures and Tables

**Table 1:** Data for borazine combustion experiments

Expt. No.	HNO_3_ titrated	H_3_BO_3_ titrated	H_3_BO_3_ titr. H_3_BO_3_ calc.	BN	Δ*e*	*E_a_*	Δ*Rc*	*q_i_*	*q_n_*	W.C.	*q*_BN_	*S*	−Δ*E*°(28°C)
													
	*Mole*	*Mole*		*Mole*	*j/ohm*	*j/ohm*	*Ohm*	*j*	*j*	*j*	*j*	*Gram*	*kj/mole*
1	0.000980	0.02016	0.9920	0.000163	16.2	141,731.3	0.109885	1 0.0	56.6	2 6.1	67.6	0.54555	2301.52
2	.000935	.02662	.9818	.000493	34.6	141,749.7	.145888	36.3	54.0	8.8	204.6	.72782	2301.79
3	.001073	.02362	.9885	.000274	41.7	141,756.8	.130025	38.2	62.0	2 7.8	113.7	.64141	2316.90
4	.001567	.03452	.9866	.000468	43.4	^3^ 141,726.8	.189172	37.1	90.6	11.5	194.2	.93923	2305.51
5	.000996	.02745	.9850	.000419	34.1	141,749.2	.150439	37.0	57.6	9.1	173.9	.74811	2305.05
6	.000878	.01809	.9904	.000175	38.5	141,753.6	.099485	37.5	50.7	6.1	72.6	.49030	2314.75
7	.000838	.01908	.9856	.000278	32.5	141,747.6	.104598	37.4	48.4	6.4	115.4	.51966	2303.26
8	.000749	.02308	.9656	.000821	41.6	141,756.7	.128189	37.4	43.3	7.8	340.7	.64159	2314.50
	
Mean													2307.91
Standard Deviation of the Mean													± 2.26

1 Platinum wire used for ignition.

2 Includes a small correction to the isothermal reaction for deviation of the final temperature from 28° C. Used *E*_8_ = 141,683.4 ± 10.4 j/ohm for a 2° temperature rise.

## Footnotes

1 Figures in brackets indicate literature references at the end of this paper.

2 Determined from freezing-point measurements by the Pure Substances Section of the Chemistry Division.

3 Made by the Constitution and Microstructure Section of the Mineral Products Division.
